# Ganoderma Lucidum Polysaccharides Enhance the Abscopal Effect of Photothermal Therapy in Hepatoma-Bearing Mice Through Immunomodulatory, Anti-Proliferative, Pro-Apoptotic and Anti-Angiogenic

**DOI:** 10.3389/fphar.2021.648708

**Published:** 2021-07-06

**Authors:** Qing-Hai Xia, Cui-Tao Lu, Meng-Qi Tong, Meng Yue, Rui Chen, De-Li Zhuge, Qing Yao, He-Lin Xu, Ying-Zheng Zhao

**Affiliations:** Department of Pharmaceutics, School of Pharmaceutical Sciences, Wenzhou Medical University, Wenzhou City, China

**Keywords:** ganoderma lucidum polysaccharides, photothermal immunotherapy, abscopal effect, hepatoma, immunomodulatory, anti-proliferative, pro-apoptotic, anti-angiogenic

## Abstract

Hepatocellular carcinoma is a malignant tumor with high morbidity and mortality, a highly effective treatment with low side effects and tolerance is needed. Photothermal immunotherapy is a promising treatment combining photothermal therapy (PTT) and immunotherapy. PTT induces the release of tumor-associated antigens by ablating tumor and Ganoderma lucidum polysaccharides (GLP) enhance the antitumor immunity. Results showed that Indocyanine Green (ICG) was successfully encapsulated into SF-Gel. ICG could convert light to heat and SF-Gel accelerates the photothermal effect *in vitro* and *in vivo*. PTT based on ICG/ICG-SF-Gel inhibited the growth of primary and distal tumors, GLP enhanced the inhibitory efficacy. ICG/ICG-SF-Gel-based PTT and GLP immunotherapy improved the survival time. ICG/ICG-SF-Gel-based PTT induces tumor necrosis and GLP enhanced the photothermal efficacy. ICG/ICG-SF-Gel-based PTT inhibited cell proliferation and angiogenesis, induced cell apoptosis, enhanced cellular immunity, and GLP enhanced these effects. In conclusion, GLP could enhance the abscopal effect of PTT in Hepatoma-bearing mice.

## Introduction

Primary liver cancer is one of the malignant tumors with high morbidity and mortality in the world. In 2018, Liver cancer ranks 6th in new incidence and 4th in mortality in the world, and the overall 5-years survival rate is only 18% (Bray et al., 2018). Hepatocellular carcinoma (HCC) is the main type of primary liver cancer (75–85%). In 2019, HCC ranks 4th in the incidence and 2nd in mortality among malignant tumors in China (Rongshou et al., 2019).

Surgical resection is the main radical treatment for HCC. Due to the late stage of the disease, poor liver function, poor general condition, only 20% of patients can be diagnosed with radical treatment. Radiofrequency ablation, hepatic arterial chemoembolization, systemic therapy and radiotherapy are available for patients who can’t or refuse surgery. However, these treatments have problems such as high side effects and tolerance. The 5-years overall survival rate of surgical resection is only 50%, and the recurrence rate is 70% (Tabrizian et al., 2015). There is no effective treatment, resulting in few options for patients with recurrence and advanced stage. Therefore, highly effective cancer treatment is urgently needed.

In recent years, cancer immunotherapy that stimulates the immune system to attack tumors has gradually become a new strategy (Kun, 2015; Ribas and Wolchok, 2018). Immunotherapy is divided into Targeted antibodies (Sliwkowski and Mellman, 2013; Compte et al., 2018), adoptive cell therapies (Restifo et al., 2012; Maude et al., 2014), oncolytic viruses (Twumasi-Boateng et al., 2018), cancer vaccines (Carreno et al., 2015; Zhu et al., 2017), and immunomodulators (Gubin et al., 2014; Momin et al., 2019). However, most immunotherapies have limitations such as high cost (Ledford, 2013; Ledford, 2015), cytokine release syndrome (DeFrancesco, 2014), risk of serious autoimmune diseases (Montero et al., 2007), delayed effect of curative effect (Hoos, 2007), and poor Chimeric antigen receptor T Cell persistence or cancer cell resistance (Shah and Fry, 2019).

Among these immunotherapies, cancer vaccine therapy may have advantages (Fang et al., 2014; Palucka and Banchereau, 2014). Tumor antigens are introduced into the body and activate B and T cells to recognize and act on specific tumor cells, to inhibit the growth, metastasis, and recurrence of tumors. In recent years, tumor vaccines have been widely studied, and whole-cell or cell lysate vaccine shows a good application prospect (Ellebaek et al., 2012; Fang et al., 2014). However, the clinical efficacy of whole-cancer vaccines is poor, and there are problems such as the complex production process, the uncertainty of nature and dose (Cicchelero et al., 2014). Therefore, more effective tumor immunotherapy is urgently needed.

PTT utilize the heat generated by optical absorbing agents under near-infrared (NIR) light to dissolve tumor. PTT has the advantages of local treatment, high selectivity, low systemic toxicity, non-invasive and controllable temperature (Chen et al., 2014). Indocyanine green (ICG) is a safe, effective, and widely used clinical contrast agent, which was approved by the United States Food and Drug Administration in 1959. Because of its NIR optical properties, ICG absorbs light and generates heat for the ablation of the tumor (Deng et al., 2015). Hyperthermia can be effective for local cancer treatment due to the sensitivity of tumor cells to temperature elevation (Hynynen and Lulu, 1990). However, it’s difficult to completely eradicate the tumor by PTT alone, heterogeneous heat distribution may lead to residual tumors (You et al., 2012). Therefore, successful cancer treatment requires the combination of PTT with other therapies, such as immune stimulation.

Photothermal immunotherapy is a promising treatment combining PTT and immunotherapy (Yata et al., 2017). PTT induces apoptosis or necrosis of tumor cells through hyperthermia (Zhang et al., 2014), releases tumor-associated antigens, triggers specific antitumor immunity, and clearing the residual tumor (Guo et al., 2014). However, weak immunogenicity affects the cancer immunotherapy effects (Zhou et al., 2012). Immunomodulators can activate the antitumor immune response and enhance immune function. Ganoderma lucidum polysaccharides (GLP) is one of the critical bioactive components of Ganoderma lucidum, which has been recognized as a promising natural source of immunomodulatory (Wang et al., 2018). Clinical studies have shown beneficial effects of GLP as an immunomodulatory in cancer patients without obvious toxicity and GLP exerts the antitumor action by stimulating the immune function (Sohretoglu and Huang, 2018). It was reported that GLP could activate bone marrow-derived macrophages to produce immunomodulatory substances, such as TNF-α, IL-1β and IL-6 (Wang et al., 1997; Zhang et al., 2010). The *in vitro* and *in vivo* studies have shown that GLP can promote the proliferation of splenocytes stimulated by Concanavalin A or lipopolysaccharide, enhance the phagocytosis of macrophages, increased cytotoxic T lymphocyte cytotoxicity and natural killer activity, increase the expression of IL-6 and TNF-α, and decrease the expression of VEGFA, which indicating that GLP possesses potential anticancer activity through immunomodulatory, anti-proliferative, pro-apoptotic and anti-angiogenic effects (Lin and Zhang, 2004; Gao et al., 2005; Chang et al., 2009; Weng and Yen, 2010).

Silk fibroin is an FDA-approved natural polymer extracted from Bombyx mori cocoons, has been extensively used because of its good biocompatibility, controllable biodegradability, remarkable biomechanical properties and self-assembling capacity (Yucel et al., 2014). Injectable SF hydrogel can be formed by physical and chemical methods through β-Sheet (Matsumoto et al., 2006). It can control drug release and widely used in local therapy (Rojas et al., 2019).

**FIGURE 1:**
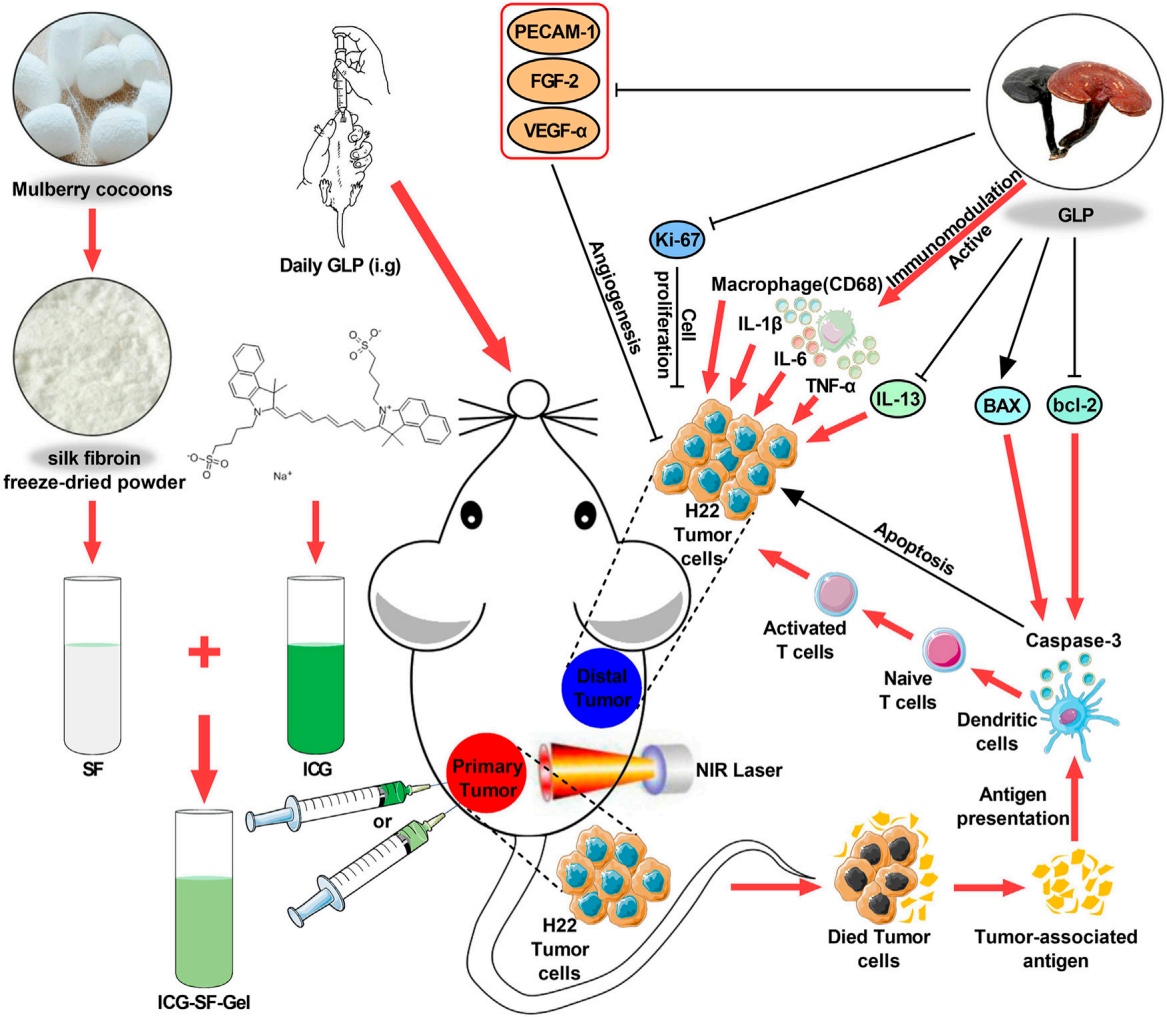
Schematic design of ICG/ICG-SF-Gel and the distal antitumor mechanism of ICG/ICG-SF-Gel-based photothermal therapy and GLP immunotherapy in hepatoma-bearing mice.; Abbreviations: SF, Silk fibroin; GLP, Ganoderma lucidum polysaccharides; ICG, Indocycline green; ICG-SF-Gel, Indocyanine green and Silk fibroin Gel; NIR, near-infrared.

In this study, SF was extracted from the Bombyx mori cocoons, ICG-SF-Gel was formed by ultrasonic oscillation method, GLP was prepared by heating and dissolving. ICG was encapsulated into SF-Gel for phototherapy of Hepatic Tumor (As illustrated in Figure 1) (Chen et al., 2016). Then, the ultraviolet-visible (UV) absorbance of ICG and ICG-SF solution and photothermal effect were carefully characterized *in vitro*. Meanwhile, we established a subcutaneous bilateral hepatic tumor model. GLP was administered by intragastric administration daily, and photothermal treatment was performed. The photothermal effect of ICG/ICG-SF-Gel on tumors was recorded, the inhibitory effect on tumor growth was monitored, the body weight was measured and the mortality of mice was recorded. The morphological changes and pathological changes of tumors were detected by Haematoxylin-eosin (HE)-staining. Finally, immunohistochemical staining was used to detect the expressions of Ki-67, Caspase-3, BAX, bcl-2, PECAM-1, VEGFA, FGF-2, TNF-α, CD68, IL-1β, IL-6 and IL-13 (Figure 4). Our study indicated that GLP could enhance the abscopal effect of PTT in Hepatoma-bearing mice through immunomodulatory, anti-proliferative, pro-apoptotic and anti-angiogenic.

## Materials and Methods

### Materials and Reagents

GLP was extracted from the fruiting body of Ganoderma lucidum (Leyss. ex Fr.) Karst by Hangzhou Johncan International, the content was about 30%. ICG was purchased from Dalian Meilun Biotech (Dalian, China). Bombyx mori Cocoons were purchased from Bozhou Naxi Pharmaceutical (Bozhou, China). Lithium Bromide was purchased from Sigma-Aldrich (St Louis, MO, United States). Dialysis bag (3,500 Da) and HE staining kit were purchased from Solarbio (Beijing, China).

### Preparation of Silk Fibroin, Indocyanine Green and Silk Fibroin Gel and Ganoderma Lucidum Polysaccharides

SF was prepared as the method previously described (Kim et al., 2017). Bombyx mori Cocoons were cut into pieces and soaked in 0.02 M Na_2_CO_3_ solution. Then boiled for 1 h to remove sericin while stirring constantly. Washed 3 times and dried at 65°C. After drying, cocoons were dissolved in a water bath with 9.3 M LiBr solution at 60°C for 3 h. Then, filtrated twice with filter paper. Finally, dialyzed with dialysis bag (MWCO 3500) against 2,000 ml ultra-pure water for 72 h to remove the LiBr. The SF solution was further purified by freeze-drying to obtain silk fibroin freeze-dried powder, sealed and stored in a drying box.

ICG-SF-Gel was prepared by ultrasonic oscillation. Briefly, ICG solution (2 mg/ml) and SF solution (60 mg/ml) were mixed in an equal proportion. Then, ultrasound (100 W) was performed until turbidity appeared. Finally, the ultrasound was stopped and standing for 30 min.

GLP was prepared by heating and dissolving. Briefly, 1 g of GLP powder was added to 6 ml of pure water, heated and dissolved in a constant temperature water bath at 60°C, centrifuged at 2,000 rpm for 5 min, then the precipitation was discarded to get about 6 ml of GLP solution.

### The Photothermal Effect of Indocyanine Green and Indocyanine Green and Silk Fibroin Gel *in vitro*

To investigate the photothermal effect of ICG and ICG-SF-Gel *in vitro*, 200 μl of ICG solution (1 mg/ml) and ICG-SF-Gel were added into a 48-well cell culture plate and irradiated with 808 nm NIR laser (1.0 W/cm^2^) for 300 s. During the irradiation, the temperature was recorded. SF (30 mg/ml) and Ultra-pure water were used as control. To investigate the photothermal circle stability, the temperature changes of four groups were measured by light on/off (5 min/30 min) three times.

### Cell Culture

Mouse hepatoma 22 (H22) cells were purchased from Shanghai Zhong Qiao Xin Zhou Biotechnology (Zqxzbio, ZQ0109, China). The cells were cultured in RMPI Medium 1,640 (Gibco, C11875500BT, United States), supplemented with 10% fetal bovine serum (Gibco, 1600044, United States), 1% penicillin, and streptomycin (Solarbio, P1400, China) at 37°C under a circumstance containing 5% CO_2_. Adjust H22 cell strains to 1 × 10^7^ cells/ml, then injected 0.2 ml into the abdomen of male BALB/C mice for 6–8 days. The ascites cells were passaged three times for usage. The cell concentration was 1 × 10^7^ cells/ml.

### Animal Experiments

#### Animals

Male BALB/C mice (20–24 g) were supplied by the Laboratory Animals Center of Wenzhou Medical University. The mice were kept with regulated humidity (50 ± 10%) and temperature (22 ± 2°C) in a 12 h light/dark cycle, fed with forage and clean water ad libitum. All the experiments were performed under the approval and guidance of the Animal Experimentation Ethics Committee of Wenzhou Medical University, Wenzhou, China.

Mice were randomly divided into seven groups. In the H22, H22 + ICG + Laser, H22 + ICG-SF-Gel + Laser and H22 + Laser groups, mice received ultra-pure water intragastric administration daily, while in the H22 + GLP, H22 + ICG + GLP + Laser and H22 + ICG-SF-Gel + GLP + Laser groups, mice received GLP (50 mg/ml) intragastric administration daily.

#### The Primary Tumor Inoculation

After 7 days of intragastric administration, 0.2 ml of the H22 cells (1 × 10^7^ cells/ml) were subcutaneously injected into the back of the left hindlimb of each mouse as primary tumors. The body weight and primary tumor volume were measured daily. Tumor volumes (V) were calculated using an ellipsoid approximation: V = 0.5 × L × W^2^ (L = the maximum diameters of the tumor, W = a longest transverse diameter perpendicular to the maximum diameters of the tumor). Relative primary tumor volumes were calculated as V/V_0_ (V_0_ is the tumor volume when ICG/ICG-SF-Gel were injected). The mortality of mice was recorded daily, draw the curve of the survival time in mice by Kaplan-Meier method.

#### The Distal Tumor Inoculation

Eight days after the primary tumor inoculation, 0.2 ml of the H22 cells (1 × 10^7^ cells/ml) were subcutaneously injected into the back of the right forelimb of each mouse as the distal tumors. The distal tumor volumes were measured daily. Tumor volumes (V) were calculated using an ellipsoid approximation: V = 0.5 × L × W^2^. Relative distal tumor volumes were calculated as V/V_0_ (V_0_ is the tumor volume of the third day after distal tumor inoculation).

#### The Photothermal Effect of Indocyanine Green and Indocyanine Green and Silk Fibroin Gel *in vivo*

When the primary tumors reached 60 mm^3^, 0.1 ml ICG (1 mg/ml) was injected into the tumor site of H22 + ICG + Laser and H22 + ICG + GLP + Laser groups, 0.1 ml ICG-SF-Gel was injected into the tumor site of H22 + ICG-SF-Gel + Laser and H22 + ICG-SF-Gel + GLP + Laser groups. After injections, the tumor site of mice in the four groups was irradiated with an 808 nm NIR laser for 90 s. The maximum temperatures of the tumor area and thermo-graphic images were recorded at every 10 s intervals for 90 s. The irradiation and temperature thermal imaging were performed again each day for the next 2 days.

### The Distal Antitumor Mechanism of Photothermal Therapy and Ganoderma Lucidum Polysaccharides Immunotherapy

#### Histopathological Examination

Four mice from each group were randomly sacrificed 7 days after the distal tumor inoculation and their primary and distal tumors were collected. Tumors were fixed in 4% paraformaldehyde and embedded in paraffin wax, cut into 5 µm thick slices. HE-stained slides were obtained for morphological and pathological analysis.

#### Immunohistochemical Staining

Tumor slices were deparaffinized in a xylene series and hydrated in distilled water. Then, Incubating slides in 3% H_2_O_2_ in methanol for 10 min. Antigen retrieval was performed by heating slides in an autoclave with 10 mM pH 6.0 citrate buffer for 5 min after pressure gaining and washing with PBS. Nonspecific antibody binding was blocked with 5% BSA in PBS for 30 min at room temperature before incubation with primary antibodies at 4°C overnight. Then, incubated with secondary antibody for 1 h at 37°C. Finally, stained with DAB and counterstained with hematoxylin. Negative control slides were obtained by omitting the primary antibody. Cell nuclei were counterstained with reformative Gill’s hematoxylin.

#### Quantitation of Ki-67 Proliferation Index

The method has been described previously. Briefly, the tumors from different treatment groups were collected for immunohistochemical staining of Ki-67. Ki-67 positive cells were counted from at least five random microscopic fields (400 × original magnifications) per subject and quantified by optical density using Image-Pro Plus 6.0. The Ki-67 proliferation index (%) was calculated according to the following formula: the number of Ki-67 positive cells/the total cell count × 100%.

#### Detection of Tumor Apoptosis, Tumor Vascularity, and Immune Index

The method has been described previously. Briefly, tumors from different treatment groups were collected for immunohistochemical staining of Caspase-3, BAX, bcl-2, PECAM-1, VEGFA, FGF-2, TNF-α, CD68, IL-1β, IL-6, and IL-13. Positive-staining cells were counted from at least five random microscopic fields (400 × original magnifications) per subject and quantified by optical density using Image-Pro Plus 6.0.

### Statistical Analysis

All the data were presented as mean ± SD. All statistical analysis was carried out using GraphPad Prism 7.04 for Windows. For all the immunohistochemical staining analysis, the Student’s t-test was performed to determine the significance of differences between two groups, and one-way analysis of variance (ANOVA) was employed for multiple group comparison. Survival curves were plotted using the Kaplan-Meier method and compared between groups using the Log-rank (Mantel-Cox) test. Two-way ANOVA was used for time-temperature curves. The temperature curve, relative tumor growth curve and body weight curve were analyzed by two-way ANOVA. In all tests, *p* < 0.05 was considered statistically significant. Statistical significance was expressed by *p < 0.05, **p < 0.01, ***p < 0.001 and ****p < 0.0001.

## Results and Discussion

### Preparation of Silk Fibroin and Indocyanine Green and Silk Fibroin Gel and Ganoderma Lucidum Polysaccharides

SF was prepared as the method previously described. The concentration of SF solution was approximately 3 wt%. ICG solution (2 mg/ml) and SF solution (60 mg/ml) were mixed in an equal proportion. Then, ultrasound (100 W) was performed until turbidity appeared. Finally, the ultrasound was stopped and standing for 30 min to obtain ICG-SF-Gel.

**FIGURE 2:**
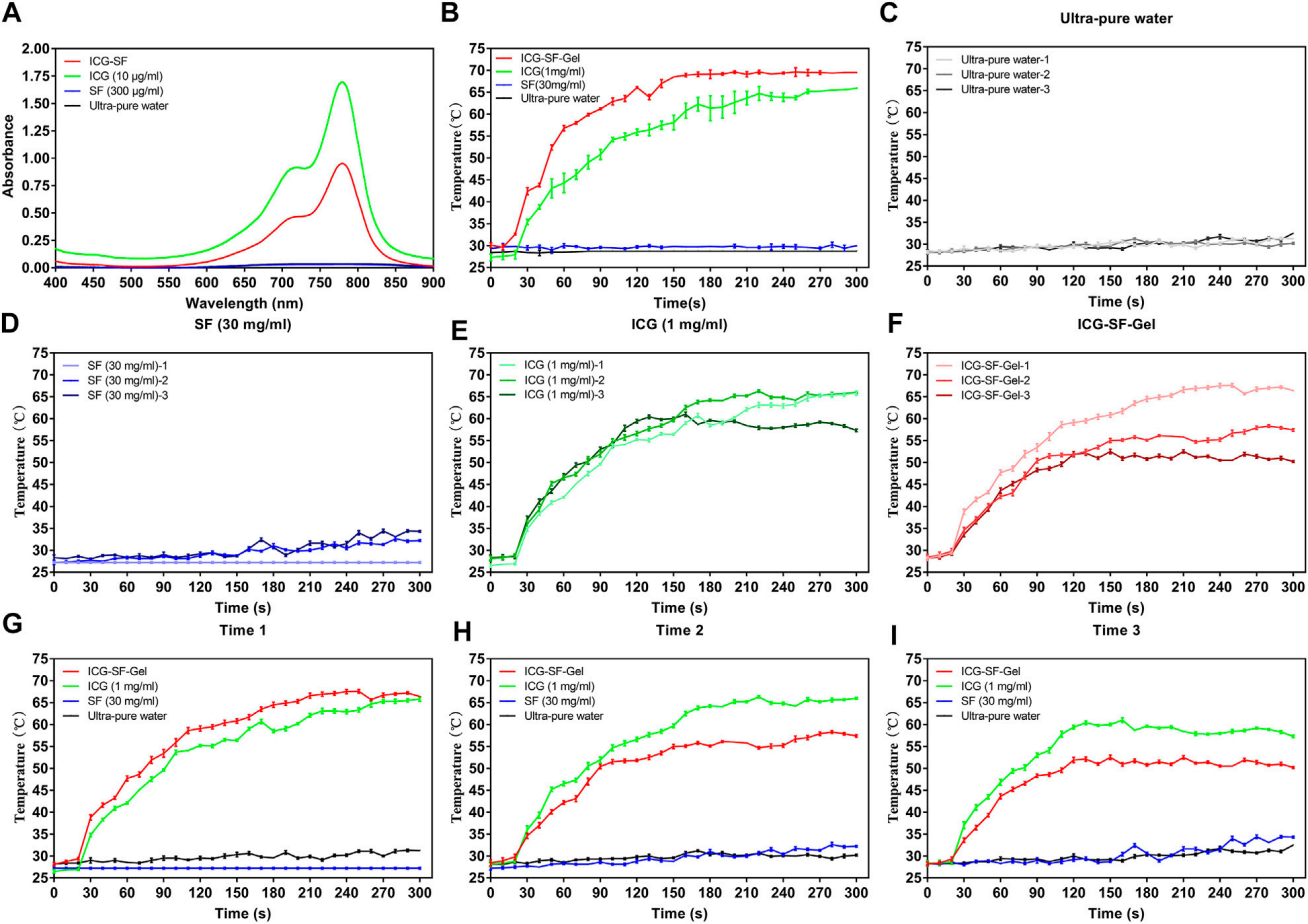
The photothermal effect of ICG and ICG-SF-Gel *in vitro*. **(A)** The ultraviolet-visible spectrum of the ICG-SF, ICG, SF, and Ultra-pure water; **(B)** The temperature change of ICG-SF-Gel, ICG, SF and Ultra-pure water under continuous 808 nm NIR laser irradiation (1 W/cm^2^) for 300 s; **(C–F)** Temperature change of Ultra-pure water **(C)**, SF **(D)**, ICG **(E)** and ICG-SF-Gel **(F)** under the discontinuous 808 nm NIR irradiation mode (ON = 5 min, OFF = 30 min, three cycles, 1 W/cm^2^) for 300 s; **(G–I)** Three cycles temperature change of Ultra-pure water, SF, ICG and ICG-SF-Gel under the discontinuous 808 nm NIR irradiation mode (ON = 5 min, OFF = 30 min, 1 W/cm^2^) for 300 s.

The UV spectrum of ICG (10 μg/ml) and ICG-SF solutions was investigated and results were displayed in Figure 2A. The Ultra-pure water and SF solution exhibited no absorption peaks at 400–900 nm. ICG and ICG-SF solutions exhibited a strong characteristic absorption peak at 779 nm, indicating that the successful encapsulation of ICG in the SF solution and SF did not affect the UV absorbance of ICG.

In our previous study, we carried out the characterization experiments of ICG-SF-Gel, which proved the safety of ICG-SF-Gel (Xu et al., 2018; Yao et al., 2020).

GLP was extracted from the fruiting body of Ganoderma lucidum (Leyss. ex Fr.) Karst by Hangzhou Johncan International, the content was about 30%. It was prepared by heating and dissolving. Briefly, 1 g of GLP powder was added to 6 ml of pure water, heated and dissolved in a constant temperature water bath at 60°C, centrifuged at 2000 rpm for 5 min, then the precipitation was discarded to get about 6 ml of GLP solution.

### The Photothermal Effect of Indocyanine Green and Indocyanine Green and Silk Fibroin Gel *in vitro*

The photothermal effect of ICG-SF-Gel, ICG (1 mg/ml) *in vitro* was evaluated for 300 s and results were shown in Figures 2B–I.

To explore a more suitable concentration of ICG, we carried out experiments on the photothermal effect of ICG at different concentrations and 1 mg/ml was selected. (Figure 2B). The result showed that the photothermal effect of the continuous 808 nm NIR radiation on Ultra-pure water and SF (30 mg/ml) was negligible. By contrast, after radiation for 10 s, the temperature of ICG (1 mg/ml) began to rise rapidly, after 260 s the temperature was elevated up to 65°C. ICG-SF-Gel also showed a temperature rising after radiation. The temperature of ICG-SF-Gel began to rise rapidly after 10 s, then rose and remained at 69°C after 160 s. Compared with the ICG solution, ICG-SF-Gel has a higher temperature and faster heating rate (Figure 2B).

To investigate the photothermal circle stability *in vitro*, the temperature change of ICG (1 mg/ml) and ICG-SF-Gel under the discontinuous 808 nm NIR irradiation mode (ON = 5 min, OFF = 30 min, three cycles, 1 W/cm^2^) was performed (Figures 2C–I) and the similar results were obtained (Figure 2G). The photothermal effect of the first radiation on the temperature of Ultra-pure water and SF (30 mg/ml) was negligible. By contrast, after radiation for 20 s, the temperature of ICG (1 mg/ml) began to rise rapidly, after 200 s the temperature was elevating up to 60°C. ICG-SF-Gel also exhibited a temperature rising after the first radiation, the temperature began to rise rapidly after 20 s, and after 140 s the temperature was elevated up to 60°C. Compared with the ICG solution, a higher temperature and faster heating rate were observed for ICG-SF-Gel (Figure 2G). As expected, the photothermal effect of discontinuous 808 nm NIR irradiation mode on the temperature of Ultra-pure water and SF (30 mg/ml) were negligible (Figures 2C,D). Compared with the ICG solution, the heating rate and maximum platform temperature of ICG-SF-Gel decreased more obviously (Figures 2E,F). In Figure 2E, the temperature rise of the three radiations was the same in the first 40 s, indicating that the photothermal stability of ICG is good. Besides, the first radiation also consumed ICG, but less than the ICG-SF-Gel group, which was still enough to support the ICG consumption of the second radiation, so the second photothermal curve did not decrease. However, in the third radiation, the content of ICG has been consumed too much, resulting in a decrease in the temperature in the third photothermal curve. In Figure 2F, SF-Gel could control the release of ICG and aggregate ICG, resulting in more ICG consumption, more temperature rise, and faster heating rate during the first radiation. Then, also due to the controlled release and aggregation effect of SF-Gel on ICG, the consumption of ICG was higher. Therefore, in the second and third laser irradiations, the content of ICG was lower, the heating rate and the maximum platform temperature of the ICG-SF-Gel group decreased more obviously. Therefore, in the follow-up animal experiments, we had an injection of ICG before every radiation to ensure the stability of the effect of PTT.

Overall, these results suggested that ICG could convert light to heat, and ICG-SF-Gel may converge and accelerate the photothermal effect so that ICG can achieve a faster and stronger photothermal effect.

### The Photothermal Effect of Indocyanine Green and Indocyanine Green and Silk Fibroin Gel *in vivo*

**FIGURE 3:**
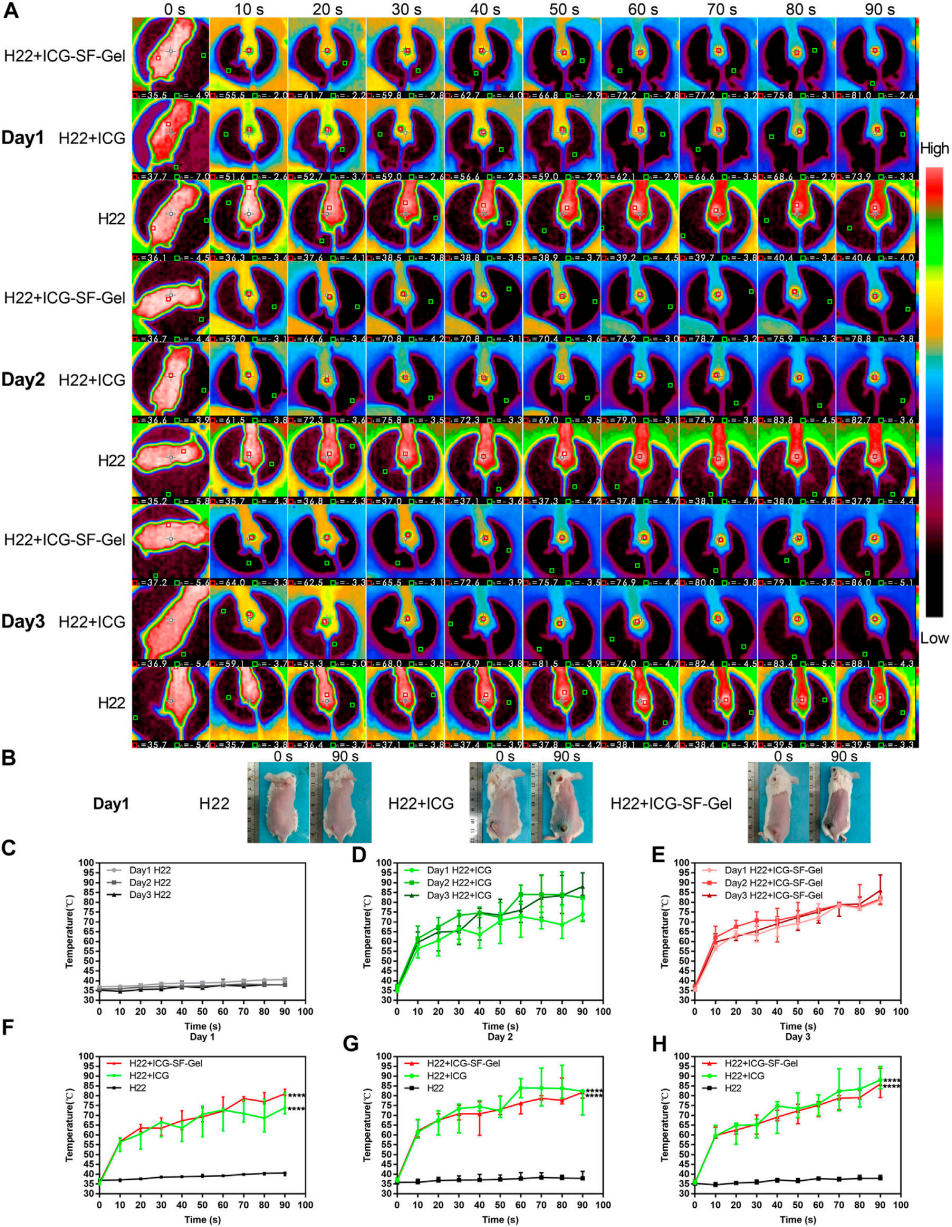
The photothermal effect of ICG and ICG-SF-Gel *in vivo*. **(A)** The thermo-graphic images of H22 + ICG-SF-Gel, H22 + ICG, and H22 groups mice after NIR irradiation at every 10 s intervals for 90 s during the 3 days NIR irradiation; **(B)** The digital photograph of H22 + ICG-SF-Gel, H22 + ICG and H22 groups mice before and after NIR irradiation on Day 1; **(C–E)** The temperature changes of tumor area in H22 **(C)**, H22 + ICG **(D)** and H22 + ICG-SF-Gel **(E)** groups mice after NIR irradiation at every 10 s intervals for 90 s during the 3 days NIR irradiation; **(F–H)** The temperature changes of tumor area in H22, H22 + ICG and H22 + ICG-SF-Gel groups mice after NIR irradiation at every 10 s intervals for 90 s on Day 1 **(F)**, Day 2 **(G)** and Day 3 **(H)** (*n* = 3 mice per group, data are presented as the mean ± SD. Statistical significance was expressed by *p < 0.05, **p < 0.01, ***p < 0.001 and ****p < 0.0001, comparing other groups with the H22 group).

To confirm the photothermal transformation of ICG/ICG-SF-Gel *in vivo*, and to stimulate the immune response of the body, we treated the mice with 808 nm NIR laser (1 W/cm^2^) every 24 h intervals for 3 days. The maximum temperatures of the tumor area and thermo-graphic images were recorded for 90 s (Figure 3A and Supplementary Material).

As shown in Figures 3C–F, on the first day, the tumor area temperature of H22 group mice increased slowly from 36.6 to 40.2°C. By contrast, the tumor area temperature of H22 + ICG group mice rapidly increased from 35.6 to 55.5°C after irradiation for 10 s, then kept increasing at a relatively fast rate. Finally, the temperature was elevated up to 75.5°C. In the ICG-SF-Gel group, the tumor area temperature rapidly increased from 35.6 to 56.8°C after 10 s, then kept increasing at a relatively fast rate. Finally, the temperature was elevated up to 81.4°C. Compared with the H22 + ICG group, the tumor area temperature of the ICG-SF-Gel group increased faster and higher, which may be due to the aggregation effect of SF-Gel on ICG, so that ICG can achieve a faster and stronger photothermal effect.

In the 3 days NIR irradiation experiment, the tumor area temperature of H22 group mice increased slowly from 36 to 40°C (Figure 3C). The heating rate of H22 + ICG group mice was faster and higher on day 2 than day 1, then decreased on day 3, but still faster and higher than day 1 (Figure 3D). The heating rate of ICG-SF-Gel group mice was faster and higher on day 2 than day 1, then decreased on day 3, but still slightly faster and higher than day 1 (Figure 3E). However, in the experiment of discontinuous NIR radiation *in vitro*, the heating rate of ICG and ICG-SF-Gel groups decreased slightly, and the maximum platform temperature kept decreasing. The reason for this difference may be the effect of skin color and tissue on NIR heat absorption. As shown in Figure 3B, after irradiation, the tumor areas of the mice were burned and the tissue moisture content decreased, making it easier to heat up. Besides, the pigmentation in the tumor area increases the absorption of light energy, thus making the thermal effect of the laser more significant.

Compared with the H22 + ICG group, the tumor area temperature of the ICG-SF-Gel group increased faster and higher on day 1, but slightly slower and lower on day 2 and day 3 (Figures 3F–H). It is consistent with results of discontinuous NIR radiation *in vitro*, which may be due to the aggregation effect of SF-Gel on ICG. Due to the higher consumption of ICG in the ICG-SF-Gel group mice on day 1, the photothermal effect of ICG-SF-Gel group mice was slightly weaker than that of H22 + ICG group mice on day 2 and day 3.

All in all, the results of *in vivo* experiments also demonstrated that ICG could convert light to heat, and ICG-SF-Gel may converge and accelerate the photothermal effect of ICG so that ICG can achieve a faster and stronger photothermal effect.

### Indocyanine Green/Indocyanine Green and Silk Fibroin Gel-Based Photothermal Therapy and Ganoderma Lucidum Polysaccharides Immunotherapy to Inhibit the Tumor Growth

**FIGURE 4:**
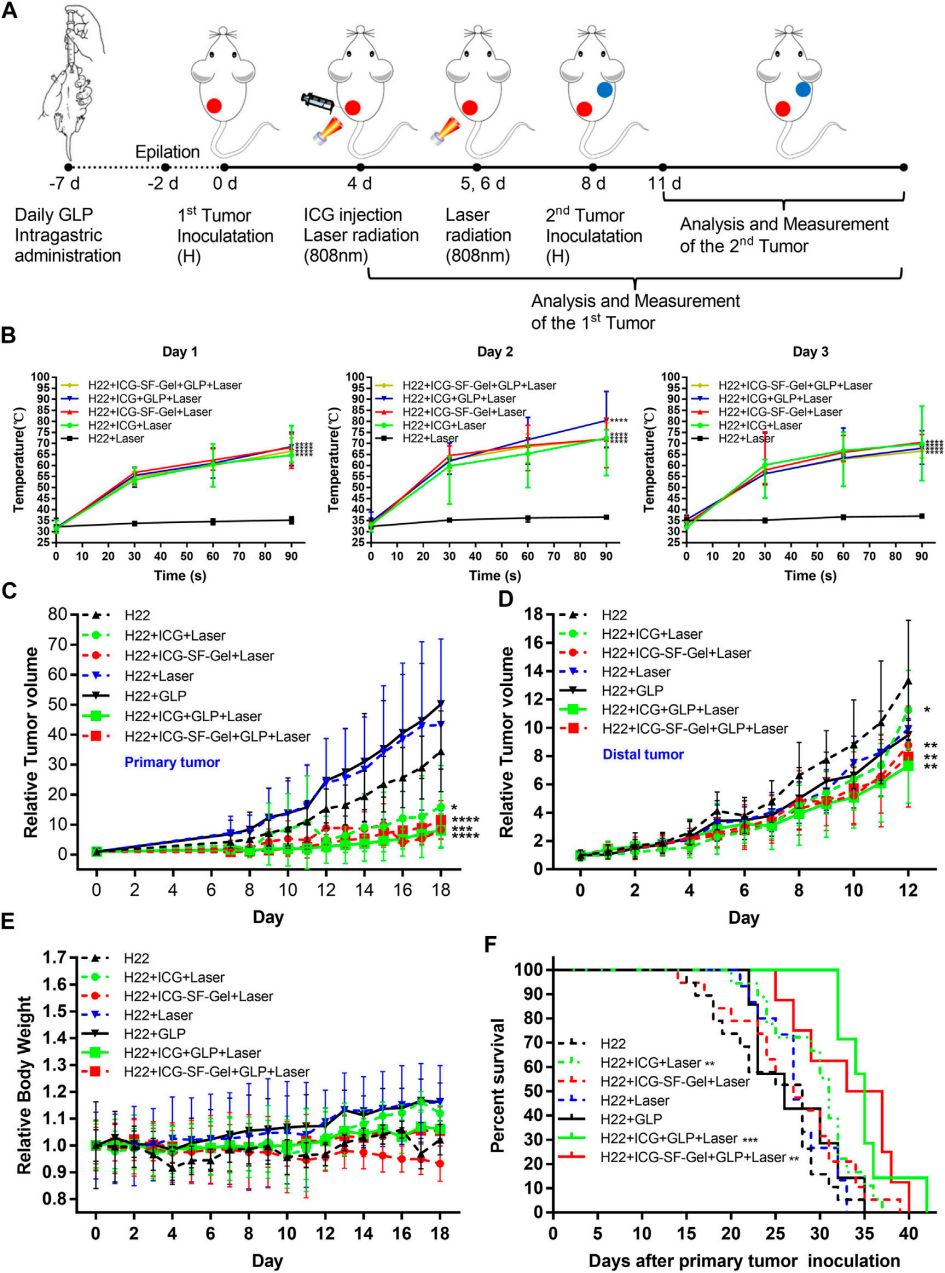
Inhibition of the tumor growth *in vivo*. **(A)** Schematic illustration of ICG/ICG-SF-Gel-based photothermal therapy and GLP immunotherapy to inhibit distal tumor growth; **(B)** The temperature changes of tumor area in five groups mice after NIR irradiation during the 3 days NIR irradiation (*n* = 6–9 mice per group, data are presented as the mean ± SD. Statistical significance was expressed by *p < 0.05, **p < 0.01, ***p < 0.001 and ****p < 0.0001, comparing other groups with the H22 group); **(C–E)** The relative tumor volume of primary tumors **(C)** or distal tumors **(D)** and the relative body weight **(E)** in seven groups mice after different treatments to their primary tumors (*n* = 10–15 mice per group, data are presented as the mean ± SD. Statistical significance was expressed by *p < 0.05, **p < 0.01, ***p < 0.001 and ****p < 0.0001, comparing other groups with the H22 group); **(F)** The survival curve of seven groups mice after primary tumor inoculation. Survival curves were compared between groups using the log-rank test (*n* = 7–19 mice per group, data are presented as the mean ± SD. Statistical significance was expressed by *p < 0.05, **p < 0.01, ***p < 0.001 and ****p < 0.0001, comparing other groups with the H22 group).

The Schematic illustration of ICG/ICG-SF-Gel-based PTT and GLP immunotherapy to inhibit distal tumor growth is shown in Figure 4A. After 7 days of ultra-pure water or GLP intragastric administration, 0.2 ml of the H22 cells (1 × 10^7^ cells/ml) were subcutaneously injected into the back of the left hindlimb of each mouse as the primary tumors. When the primary tumors reached 60 mm^3^, H22 + ICG + Laser and H22 + ICG + GLP + Laser groups were intratumorally injected with 0.1 ml ICG (1 mg/ml), H22 + ICG-SF-Gel + Laser and H22 + ICG-SF-Gel + GLP + Laser groups were intratumorally injected with 0.1 ml ICG-SF-Gel. After injections, the tumor site of mice in five groups was irradiated for 90 s. The maximum temperatures of the tumor area and thermo-graphic images were recorded for 90 s. The irradiation and temperature thermal imaging were performed again each day for the next 2 days. In the 3 days experiment, the tumor area temperature of mice injected with ICG or ICG-SF-Gel under laser irradiation quickly rose to 55°C in 30 s, which was high enough to effectively ablate tumors. While in the H22 group, the tumor area temperatures of mice exhibited no significant increase during irradiation (Figure 4B).

To investigate the antitumor efficiency of ICG/ICG-SF-Gel-based PTT and GLP immunotherapy in hepatoma-bearing mice, the primary tumor size was measured daily. The relative primary tumor volumes were calculated as V/V_0_ (V_0_ is the tumor volume when ICG/ICG-SF-Gel were injected). As illustrated in Figure 4C, ICG/ICG-SF-Gel-based PTT and GLP immunotherapy significantly inhibited the growth of the primary tumors. Compared with the H22 group, the increase of relative primary tumor volume in H22 + ICG + Laser and H22 + ICG-SF-Gel + Laser groups were slowed down significantly, while that in H22 + ICG + GLP + Laser and H22 + ICG-SF-Gel + GLP + Laser groups slowed down more obviously, suggesting that ICG/ICG-SF-Gel-based PTT inhibited the growth of primary tumors and GLP could enhance the inhibitory effect.

Besides, GLP mainly plays the role of an immunomodulator to enhance the inhibition of tumors. GLP alone can’t directly inhibit tumor growth, and GLP needs to be dissolved by heating during intragastric administration. In this process, GLP may be decomposed into glucose-based monosaccharides, which has been proved in the literature published in science that glucose can promote the growth of the primary tumor (Goncalves et al., 2019), which may lead to the relative primary tumor volume in the GLP + H22 group is higher than that in the H22 group alone. Laser alone can’t kill the H22 tumor, but due to the mild temperature stimulation caused by the laser, it accelerates the blood circulation around the tumor and promotes the growth of the primary tumor, which may lead to the relative primary tumor volume in the GLP + Laser group is higher than that in the H22 group alone (Figure 4C).

Eight days after the primary tumor inoculation, 0.2 ml of the H22 cells (1 × 10^7^ cells/ml) were subcutaneously injected into the back of the right forelimb of each mouse as the distal tumors with no direct treatments. To further investigate the distal antitumor efficiency of ICG/ICG-SF-Gel-based PTT and GLP immunotherapy in hepatoma-bearing mice, the distal tumor size was measured daily. The relative distal tumor volumes were calculated as V/V_0_ (V_0_ is the tumor volume of the third day after distal tumor inoculation). As shown in Figure 4D, ICG/ICG-SF-Gel-based PTT and GLP immunotherapy significantly inhibited the growth of distal tumors. Compared with the H22 group, the increase of relative tumor volume in H22 + ICG + Laser and H22 + ICG-SF-Gel + Laser groups were slowed down significantly, while that in H22 + ICG + GLP + Laser and H22 + ICG-SF-Gel + GLP + Laser groups slowed down more obviously, suggesting that ICG/ICG-SF-Gel-based PTT inhibited the growth of distal tumors and GLP could enhance the inhibitory efficacy.

However, unlike primary tumors, there was no significant difference in distal tumors between the H22 + GLP and H22 + laser groups and the H22 group. This may be due to the different mechanisms of combined treatment of primary and distal tumors. The inhibitory effect of combination therapy on the primary tumor is mainly the direct killing effect on tumor cells, while the inhibitory effect on the distal tumor is achieved through the distal effect brought by immune function. In the H22 + GLP group, because the effect of combination therapy on the distal tumor is achieved through immune function, the promoting effect of sugar on tumor growth may be offset by strong immune function. In the H22 + Laser group, the laser did not directly stimulate the distal tumor, so it did not affect the growth of the distal tumor.

To evaluate the health status of mice, the body weight was measured daily. The relative body weight was calibrated by normalizing the initial body weight (at day 0) to 1. The relative body weight was plotted at regular intervals and considered a surrogate for evaluation of systemic well-being. There were no significant body weight changes in the seven groups during the experiment (Figure 4E), indicating the fewer side effect of the treatments.

To evaluate the survival of mice, the mortality of mice was recorded daily. As displayed in Figure 4F, ICG-based PTT and GLP immunotherapy significantly resulted in improving the survival time. Compared with the H22 group, the H22 + ICG + Laser group exhibited a significant increase in improving the survival time, and in the H22 + ICG + GLP + Laser group, the survival time increased more obviously, indicating that ICG-based PTT prolonging the survival of mice bearing H22 tumors, and GLP can further prolong the survival time. Meanwhile, Consistent with ICG-based PTT and GLP immunotherapy, ICG-SF-Gel-based PTT and GLP immunotherapy significantly improved the survival time. Compared with the H22 group, the H22 + ICG-SF-Gel + Laser group exhibited no significant increase in improving the survival time, but in the H22 + ICG-SF-Gel + GLP + Laser group, the survival time increased significantly, indicating that ICG-SF-Gel-based PTT prolonging the survival of mice with the combination of GLP.

### The Distal Antitumor Mechanism of Indocyanine Green/Indocyanine Green and Silk Fibroin Gel-based Photothermal Therapy and Ganoderma Lucidum Polysaccharides Immunotherapy

As a promising natural source of immunomodulatory, GLP exerts the antitumor action by stimulating immune function (Sohretoglu and Huang, 2018; Wang et al., 2018). We introduced GLP immunotherapy to improve the distal antitumor efficiency of tumor-associated antigens produced *in situ* after photothermal ablation of primary tumors, and to inhibit the development of tumors.

To explore the antitumor mechanism of ICG/ICG-SF-Gel-based PTT and GLP immunotherapy, the tumors from different treatment groups of mice 7 days after the distal tumor inoculation were collected for HE staining and immunohistochemical staining.

**FIGURE 5:**
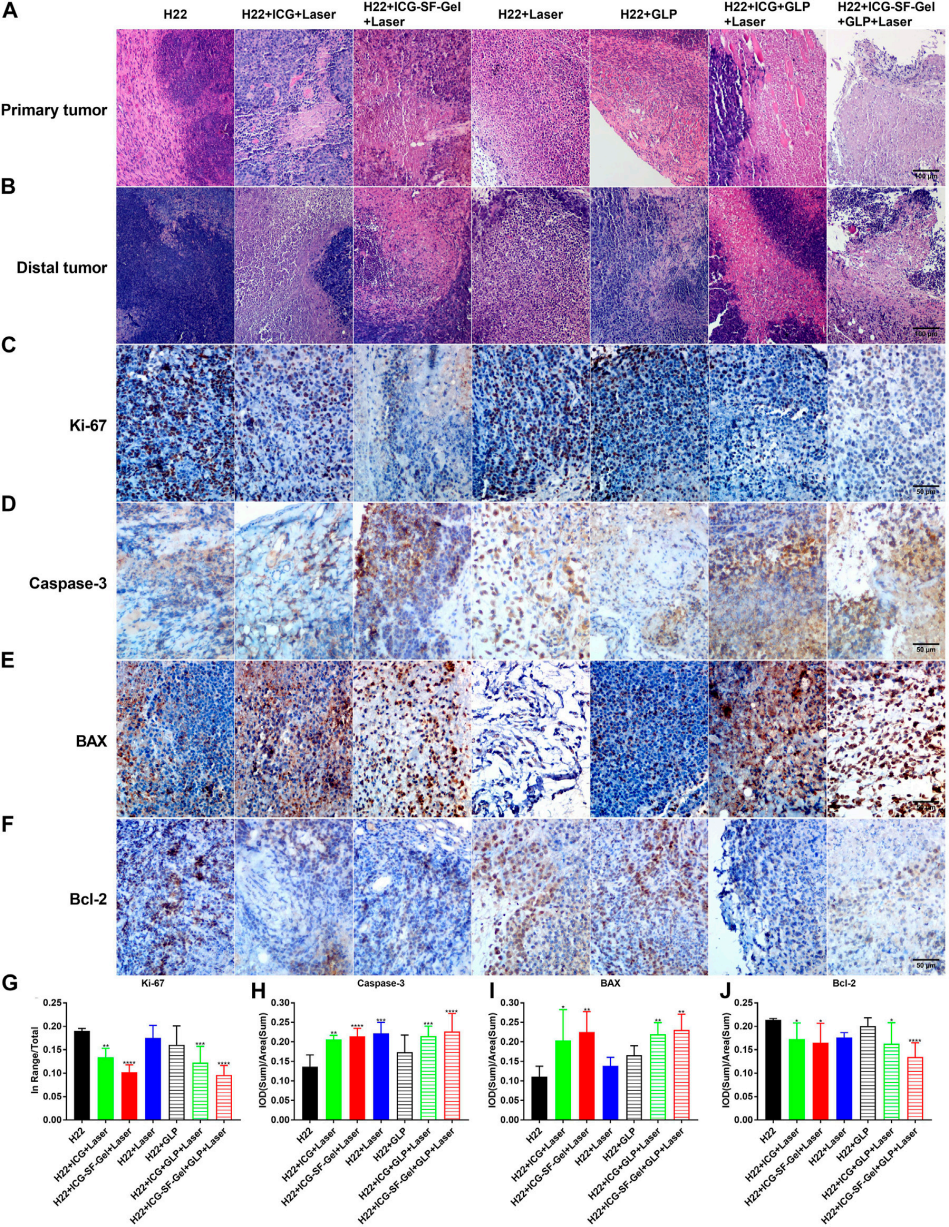
Morphological changes, pathological changes, and cell proliferation of tumor tissues after different treatments. **(A,B)** The Haematoxylin-eosin staining of primary tumors **(A)** and distal tumors **(B)** collected from different treated groups of mice 7 days after the distal tumor inoculation (The same row shared the same scale bar: 100 μm); **(C–F)** Immunohistochemical staining of Ki-67 **(C)**, Caspase-3 **(D)**, BAX **(E)** and Bcl-2 **(F)** of distal tumors collected from different treated groups of mice 7 days after the distal tumor inoculation (The same row shared the same scale bar: 50 µm) and quantitative analysis of Ki-67 **(G)**, Caspase-3 **(H)**, BAX **(I)** and Bcl-2 **(J)** (*n* = 4 mice per group, data are presented as the mean ± SD. Statistical significance was expressed by *p < 0.05, **p < 0.01, ***p < 0.001 and ****p < 0.0001, comparing other groups with the H22 group).

First, to explore the effect of photothermal ablation, the morphological and pathological changes of primary tumors from different treatment groups of mice 7 days after the distal tumor inoculation were analyzed through HE staining. As shown in Figure 5A, the H22 group displayed robust tumor tissues with no necrosis and had rich normal capillaries. Binucleated cells with large nuclei and dense chromatin were frequently observed, indicating rapid tumor growth. In contrast, cell shrinkage and nuclear fragmentation were evident in H22 + ICG + Laser, H22 + ICG-SF-Gel + Laser, H22 + ICG + GLP + Laser and H22 + ICG-SF-Gel + GLP + Laser groups, revealing that PTT based on ICG/ICG-SF-Gel could induce tumor necrosis. Besides, more tumor necrosis and fragmentation in nuclei with evident cytoplasmic separation from nuclei were found in H22 + ICG + GLP + Laser and H22 + ICG-SF-Gel + GLP + Laser groups, indicating that GLP could enhance the photothermal ablation efficacy. These results suggested that PTT based on ICG/ICG-SF-Gel could achieve the effect of photothermal ablation and GLP could enhance the photothermal ablation efficacy.

Then, the morphological changes, pathological changes, and cell proliferation of distal tumors after different treatments were analyzed through HE staining and Ki-67 immunohistochemical staining. As shown in Figure 5B, the H22 group had large areas of confluent tumor cells with little or no tumor tissue necrosis. Consistent with the primary tumors, cell shrinkage and nuclear fragmentation were observed in distal tumors after PTT based on ICG/ICG-SF-Gel, and GLP-assisted PTT led to more tumor necrosis. Besides, compared with ICG-based PTT, PTT based on ICG-SF-Gel led to more tumor necrosis, indicating that PTT based on ICG-SF-Gel have a stronger inhibitory effect on distal tumor growth than ICG-based PTT. Ki-67 is a nuclear protein expressed in proliferating mammalian cells, suggesting the high proliferation potential of the tumor cells (Sobecki et al., 2016). Ki-67 immunohistochemical staining results revealed that PTT based on ICG/ICG-SF-Gel inhibited the cell proliferation of distal tumors and GLP enhanced the inhibition effect. Compared with ICG-based PTT, ICG-SF-Gel-based PTT enhanced the inhibition of cell proliferation (Figures 5C,G). All these results suggested that PTT based on ICG/ICG-SF-Gel could inhibit the cell proliferation of distal tumors and GLP could enhance the inhibition efficacy.

Some studies have revealed that GLP reduces the expression of some signaling molecules in the phosphoinositide 3-kinase (PI3K)/AKT/mammalian target of rapamycin (mTOR) signaling pathways which play a key regulatory function in proliferation at both gene and protein levels (Suarez-Arroyo et al., 2013). In the follow-up work, we will explore whether this signaling pathway is involved in our study.

Caspase-3 is a key executioner in apoptosis which is involved in the growth stimulation (Huang et al., 2011). BAX is a pro-apoptotic gene, it has been proposed that GLP enhances the anti-cancer effects by up-regulation of BAX (Huang et al., 2010). Bcl-2 is a proto-oncogene, which can inhibit apoptosis, mainly because bcl-2 regulates a variety of cell apoptosis-related protein activity, such as by Caspase-3 (Ola et al., 2011). It was reported that GLP induced HUVECs apoptosis directly by decreasing anti-apoptotic protein Bcl-2 expression (Cao and Lin, 2006). It is recognized that GLP can induce apoptosis in cancer cells by regulating the expression of bcl-2 (Martínez-Montemayor et al., 2011). To explore whether cell apoptosis plays a role in ICG/ICG-SF-Gel-based PTT and GLP immunotherapy, the distal tumors from different treatment groups of mice 7 days after the inoculation of the distal tumor were analyzed through immunohistochemical staining of Caspase-3, BAX, and bcl-2. Compared with the H22 group, distal tumors treated with PTT based on ICG/ICG-SF-Gel displayed a more obvious expression of Caspase-3 and BAX, and GLP increased the expression, demonstrated that GLP could increase cell apoptosis of distal tumors induced by PTT based on ICG/ICG-SF-Gel. Compared with ICG-based PTT, ICG-SF-Gel-based PTT increased the expression of Caspase-3 and BAX, indicating that ICG-SF-Gel-based PTT could induce cell apoptosis of distal tumor more effectively than ICG-based PTT (Figures 5D,E,H,I). Compared with the H22 group, distal tumors treated with PTT based on ICG/ICG-SF-Gel displayed lower expression of bcl-2 and GLP decreased the expression, demonstrated that PTT based on ICG/ICG-SF-Gel could inhibit the expression of apoptosis inhibitor gene and GLP could enhance the inhibitory effect. Compared with ICG-based PTT, ICG-SF-Gel-based PTT decreased the expression of bcl-2, indicating that ICG-SF-Gel-based PTT could inhibit the expression of apoptosis inhibitor genes more effectively than ICG-based PTT (Figures 5F,J). GLP may up-regulate the expression of BAX, inhibit the expression of Bcl-2, and then activate the expression of Caspase-3, thus enhancing the apoptosis of distal tumors. All in all, these results pointed out that ICG-SF-Gel-based PTT could increase cell apoptosis of distal tumors more effectively than ICG-based PTT, and GLP could enhance the pro-apoptosis efficacy.

**FIGURE 6:**
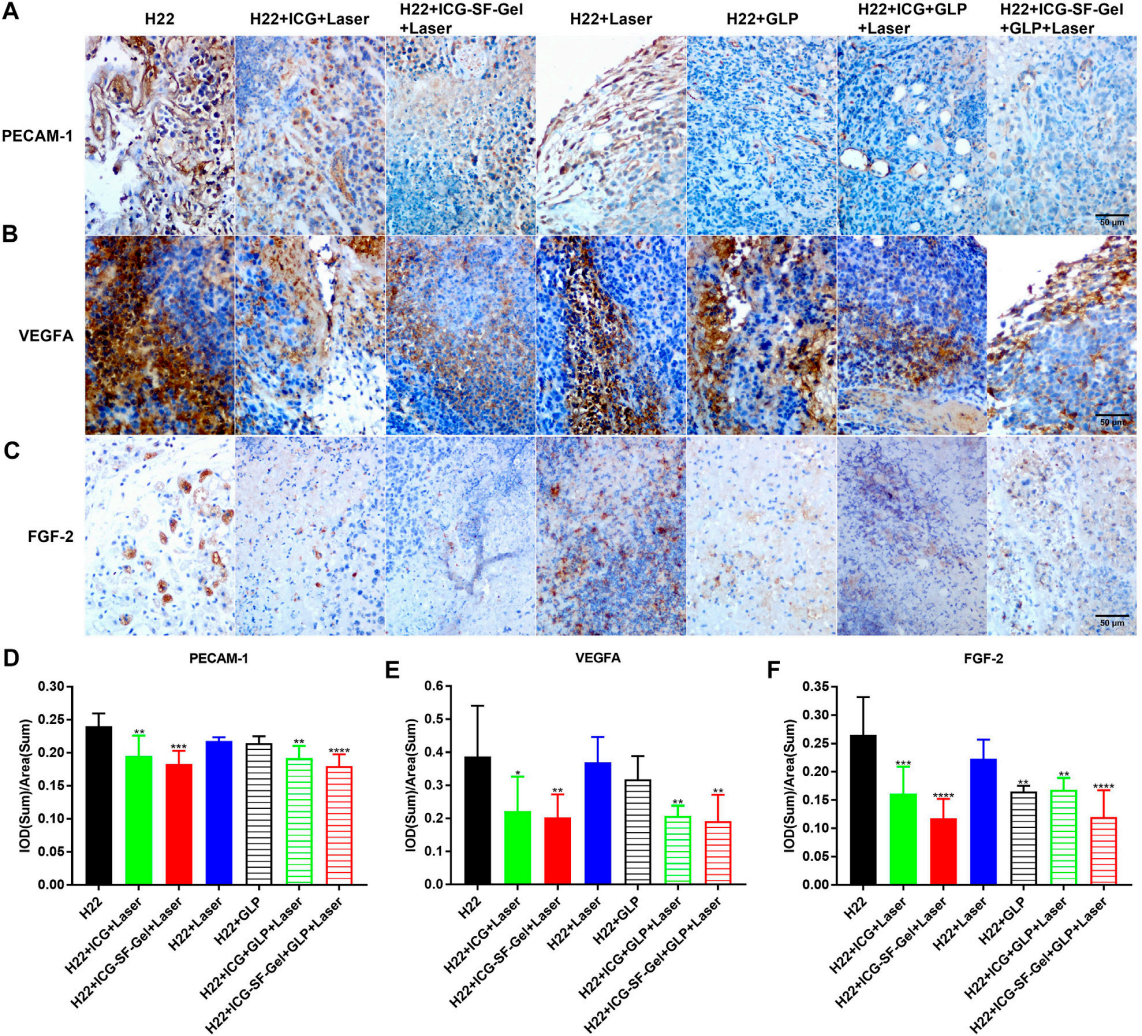
The angiogenesis of distal tumors after different treatments. **(A–C)** Immunohistochemical staining of PECAM-1 **(A)**, VEGFA **(B)** and FGF-2 **(C)** of distal tumors collected from different treated groups of mice 7 days after the distal tumor inoculation (The same row shared the same scale bar: 50 µm) and quantitative analysis of PECAM-1 **(D)**, VEGFA **(E)** and FGF-2 **(F)** (*n* = 4 mice per group, data are presented as the mean ± SD. Statistical significance was expressed by *p < 0.05, **p < 0.01, ***p < 0.001 and ****p < 0.0001, comparing other groups with the H22 group).

Platelet endothelial cell adhesion molecule-1 (PECAM-1) is a transmembrane glycoprotein member expressed on endothelial cells, which has been shown to play an important role in angiogenesis (Cao et al., 2009). Fibroblast growth factor-2 (FGF-2) and vascular endothelial growth factor (VEGF) were also involved in angiogenesis, and FGF-2 was reported to stimulate endothelial cell growth and enhances angiogenesis (Bikfalvi, 1999). Besides, GLP has been reported to inhibit angiogenesis by reducing the expression of VEGF (Sohretoglu and Huang, 2018). Therefore, angiogenesis of distal tumors from different treatment groups of mice 7 days after the inoculation of the distal tumor was analyzed through immunohistochemical staining of PECAM-1, VEGFA, and FGF-2. Compared with the H22 group, distal tumors treated with PTT based on ICG/ICG-SF-Gel displayed lower expression of PECAM-1 and VEGFA, and GLP decreased the expression more obviously, suggested that PTT based on ICG/ICG-SF-Gel could inhibit angiogenesis and GLP could enhance the inhibitory efficacy (Figures 6A,B,D,E). Compared with ICG-based PTT, ICG-SF-Gel-based PTT decreased the expression of PECAM-1, VEGFA, and FGF-2, indicating that ICG-SF-Gel-based PTT could inhibit angiogenesis more effectively than ICG-based PTT. (Figures 6A–F). These results demonstrated that ICG-SF-Gel-based PTT could inhibit angiogenesis more effectively than ICG-based PTT, and GLP could enhance the anti-angiogenic efficacy. GLP may inhibit angiogenesis by inhibiting the proliferation of vascular endothelial cells and the production of PECAM-1, VEGFA and FGF-2, thus inhibiting the growth of distal tumors.

It has been established that the immune system plays an important role in tumorigenesis (Baxevanis et al., 2009), and photothermal immunotherapy is a promising treatment combining PTT and immunotherapy (Yata et al., 2017). PTT utilizes heat generated by optical absorbing agents under NIR light to dissolve tumor cells, induced apoptosis or necrosis of tumor cells based on hyperthermia (Zhang et al., 2014). Then, tumor cell necrosis releases tumor-associated antigens, triggering specific antitumor immunity and clearing the residual tumor (Guo et al., 2014). As a promising natural source of immunomodulatory, GLP activates the antitumor immune response and enhances immune function (Sohretoglu and Huang, 2018; Wang et al., 2018). TNF-α is an important marker in the activation of cellular immunity (Xiang et al., 2015), CD68 is a pan-marker of macrophages, IL-1β and IL-6 are important proinflammatory cytokines secreted by M1 macrophages (Zong et al., 2019). IL-13 is a Th2 cytokine that plays a critical role in a novel immunoregulatory pathway in which natural killer T cells suppress tumor immunosurveillance (Terabe et al., 2000; Terabe et al., 2004). It was reported that GLP could activate bone marrow-derived macrophages to produce immunomodulatory substances, such as TNF-α, IL-1β and IL-6 (Wang et al., 1997; Zhang et al., 2010).

**FIGURE 7:**
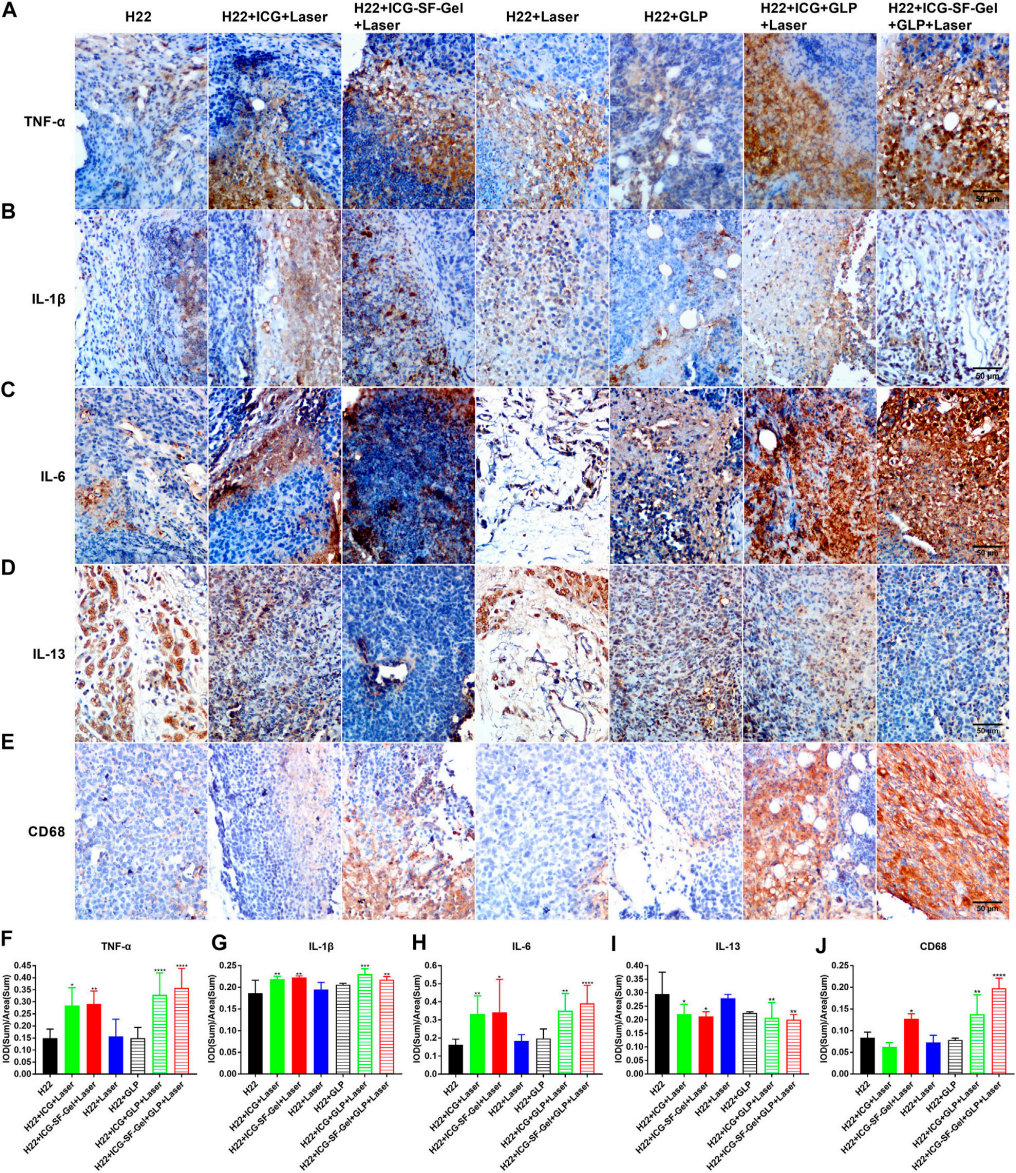
The immune index of distal tumors after different treatments. **(A–E)** Immunohistochemical staining of TNF-α **(A)**, IL-1β **(B)**, IL-6 **(C)**, IL-13 **(D)** and CD68 **(E)** of distal tumors collected from different treated groups of mice 7 days after the distal tumor inoculation (The same row shared the same scale bar: 50 µm) and quantitative analysis of TNF-α **(F)**, IL-1β **(G)**, IL-6 **(H)**, IL-13 **(I)** and CD68 **(J)** (*n* = 4 mice per group, data are presented as the mean ± SD. Statistical significance was expressed by *p < 0.05, **p < 0.01, ***p < 0.001 and ****p < 0.0001, comparing other groups with the H22 group).

To investigate whether the immune system plays an important role in ICG/ICG-SF-Gel-based PTT and GLP immunotherapy, the distal tumors from different treatment groups of mice 7 days after the distal tumor inoculation were analyzed through immunohistochemical staining of TNF-α, CD68, IL-1β, IL-6 and IL-13. Compared with the H22 group, distal tumors treated with PTT based on ICG/ICG-SF-Gel displayed a more obvious expression of TNF-α and GLP increased expression, suggesting that PTT based on ICG/ICG-SF-Gel could increase cellular immunity and GLP could enhance cellular immunity induced by PTT based on ICG/ICG-SF-Gel. Compared with ICG-based PTT, ICG-SF-Gel-based PTT increased the expression of TNF-α, indicating that ICG-SF-Gel-based PTT could induce cellular immunity more effectively than ICG-based PTT (Figures 7A,F). Compared with the H22 group, distal tumors treated with PTT based on ICG-SF-Gel displayed a more obvious expression of CD68 and GLP increased the expression of CD68 induced by ICG/ICG-SF-Gel based PTT, indicating that PTT based on ICG-SF-Gel could increase the infiltration of macrophages and GLP could enhance the infiltration of macrophages induced by ICG/ICG-SF-Gel based PTT (Figures 7E,J). Compared with the H22 group, distal tumors treated with PTT based on ICG/ICG-SF-Gel displayed a more obvious expression of IL-1β and GLP increased the expression of IL-1β induced by ICG-based PTT, indicating that PTT based on ICG/ICG-SF-Gel could increase the infiltration of M1 type macrophages and GLP could enhance the infiltration of M1 type macrophages induced by ICG-based PTT (Figures 7B,G). Compared with the H22 group, distal tumors treated with PTT based on ICG/ICG-SF-Gel displayed a more obvious expression of IL-6 and GLP increased expression, suggesting that PTT based on ICG/ICG-SF-Gel could increase the infiltration of M1 type macrophages and GLP could enhance the infiltration of M1 type macrophages induced by PTT based on ICG/ICG-SF-Gel (Figures 7C,H). Compared with the H22 group, distal tumors treated with PTT based on ICG/ICG-SF-Gel decreased the expression of IL-13 and GLP decreased the expression more obviously, indicating that PTT based on ICG/ICG-SF-Gel could inhibit the down-regulation of tumor immunosurveillance induced by IL-13 and GLP could enhance the inhibitory efficacy (Figures 7D,I). All these results suggested that the immune system plays an important role in ICG/ICG-SF-Gel-based PTT and GLP immunotherapy and GLP could enhance immune function. GLP may promote the secretion of IL-6, IL-1β and TNF-α by activating CD68 macrophages, reduce the inhibition of IL-13 secreted by natural killer T cells on tumor immune surveillance, strengthen the immune function of the body, and thus inhibit the growth of distal tumors.

Due to COVID-19 and other reasons, our experimental animals and cells suffered severe losses, which limited our research methods to immunohistochemical staining, unable to use other research methods to support the experimental results. Therefore, In the follow-up work, we will use more experimental methods to support the experimental results and conduct further research.

## Conclusion

In summary, we extracted SF from the Bombyx mori cocoons, prepared ICG-SF-Gel by ultrasonic oscillation and prepared GLP by heating dissolution. The UV absorbance of ICG and ICG-SF solution indicating that the successful encapsulation of ICG in SF solution and SF did not affect the UV absorbance of ICG. *In vitro* results showed that ICG could convert light to heat and ICG-SF-Gel may converge and accelerate the photothermal effect. To confirm the photothermal transformation of ICG and ICG-SF-Gel *in vivo*, we established a subcutaneous bilateral hepatic tumor model and shown a similar result. To investigate the antitumor efficiency, the primary and distal tumor growth rate and the mortality of mice were recorded. Results showed that PTT based on ICG/ICG-SF-Gel inhibited the growth of primary tumors and GLP could enhance the photothermal ablation efficacy. ICG-based PTT inhibited the growth of distal tumors and GLP could enhance the inhibitory effect. ICG-SF-Gel-based PTT had a stronger inhibitory effect on tumor growth than ICG-based PTT. ICG-based PTT and GLP therapy significantly resulted in improving the survival time. ICG-SF-Gel-based PTT with GLP can improve the survival time. And there were no significant body weight changes in the seven groups, indicating the fewer side effect of the treatments. To explore the distal antitumor mechanism of ICG/ICG-SF-Gel-based PTT and GLP immunotherapy, the distal tumors from different treatments were collected for HE staining and immunohistochemical staining of Ki-67, Caspase-3, BAX, bcl-2, PECAM-1, VEGFA, FGF-2, TNF-α, CD68, IL-1β, IL-6 and IL-13. Results showed that ICG/ICG-SF-Gel-based PTT induce tumor necrosis and GLP enhanced the photothermal efficacy. ICG/ICG-SF-Gel-based PTT inhibited cell proliferation and angiogenesis, induced cell apoptosis, enhanced cellular immunity, and GLP enhanced these effects. All in all, our results demonstrated that GLP could enhance the distal antitumor effect of PTT in hepatoma-bearing mice through immunomodulatory, anti-proliferative, pro-apoptotic and anti-angiogenic. The combination of PTT and immunomodulator GLP immunotherapy is potential photothermal immunotherapy on HCC.

## Data Availability

The raw data supporting the conclusion of this article will be made available by the authors, without undue reservation.

## Abbreviations

CAR, Chimeric Antigen Receptor; GLP, Ganoderma lucidum polysaccharides; HCC, Hepatocellular carcinoma; H22, Hepatoma 22; HE, Haematoxylin-eosin; ICG, Indocycline green; ICG-SF-Gel, Indocyanine green and Silk fibroin Gel; IPP, Image-Pro plus; NIR, Near-infrared; PI3K, Phosphoinositide 3-kinase; PTT, Photothermal therapy; SF, Silk fibroin; UV, Ultraviolet-visible.
